# Support Detection for SAR Tomographic Reconstructions from Compressive Measurements

**DOI:** 10.1155/2015/949807

**Published:** 2015-10-01

**Authors:** Alessandra Budillon, Gilda Schirinzi

**Affiliations:** Dipartimento di Ingegneria, Università degli Studi di Napoli “Parthenope”, Centro Direzionale di Napoli, Isola C4, 80143 Napoli, Italy

## Abstract

The problem of detecting and locating multiple scatterers in multibaseline Synthetic Aperture Radar (SAR) tomography, starting from compressive measurements and applying support detection techniques, is addressed. Different approaches based on the detection of the support set of the unknown sparse vector, that is, of the position of the nonzero elements in the unknown sparse vector, are analyzed. Support detection techniques have already proved to allow a reduction in the number of measurements required for obtaining a reliable solution. In this paper, a support detection method, based on a Generalized Likelihood Ratio Test (Sup-GLRT), is proposed and compared with the SequOMP method, in terms of probability of detection achievable with a given probability of false alarm and for different numbers of measurements.

## 1. Introduction

Synthetic Aperture Radar (SAR) tomography exploits a stack of complex-valued SAR images, acquired with different view angle and at different times, for providing the fully 3D scene reflectivity profile along azimuth, range, and elevation directions [1]. Moreover, it can also provide the 3D profile variations in time (4D SAR tomography) [2]. Then, SAR tomography allows discriminating among multiple coherent scatterers lying in the same range azimuth resolution cell and located at different elevations.

In [3–5] 3D SAR tomographic techniques, capable of achieving an increased elevation resolution, and based on compressive sampling (CS), have been proposed. These techniques exploit the sparsity assumption of the ground reflectivity profile in the elevation direction. This assumption is always met when the dominant scattering mechanism is surface scattering, like what happens in urban and scarcely vegetated areas. For highly vegetated scenes, instead, volumetric scattering is dominant, so that sparsity assumption is not verified.

CS theory enables the reconstruction of sparse or compressible signals from a small set of linear measurements. If properly chosen, the number of measurements can be much smaller than the number of Nyquist rate samples. Then, CS based techniques have been proved to be very effective for reducing the number of SAR images to be acquired and mitigating the effects due to nonuniform baseline spacing [4]. Moreover, they allow attaining superresolution reconstructions along the elevation direction [4, 5].

Nevertheless, several issues have still to be considered when dealing with 3D reflectivity profile reconstruction by means of CS based approaches. A first problem is the presence of outliers, produced by the presence of partially coherent clutter and noise and/or by possible solution instabilities. In addition, the so-called off-grid effect [6] can significantly impair the scatterers detection and localization performance [6, 7].

Another issue to be considered is that many tomographic applications do not require a full reconstruction of the signal. We are often interested only in the localization of multiple coherent scatterers and not in their intensity. This amounts in solving a sort of detection problem, dealing with the identification of only the position of the nonzero elements in the sparse unknown vector, whereas the full reconstruction of the sparse signal is not required.

In [8], it is shown that CS provides a useful framework in the development of methods for identifying the position of the nonzero elements, without fully reconstructing the signal itself. These methods are commonly referred to as “support detection” [9] and demand fewer measurements with respect to the ones based on the full reconstruction of the sparse signal.

Recently, a Generalized Likelihood Ratio Test (Sup-GLRT), searching for the best support of the unknown signal matching the data, has been introduced [10] in SAR tomography. Its detection performance can be evaluated in terms of probability of detection and probability of false alarm. If compared to classical GLRT approaches [11], it achieves better performance when the number of measurements decreases and allows the reconstruction of the unknown elevation reflectivity profile at superresolution. With respect to other methods acting directly on CS reconstruction [7, 12], it has the advantage of enabling a Constant False Alarm Rate (CFAR) approach.

In this paper, the performance of Sup-GLRT [10] is compared with the SequOMP support detection presented in [9], in terms of probabilities of detection and of false alarm. The compressive measurement capability is evaluated by analyzing the detection performance when decreasing the number of measurements.

## 2. The Signal Model

**Figure 1:**
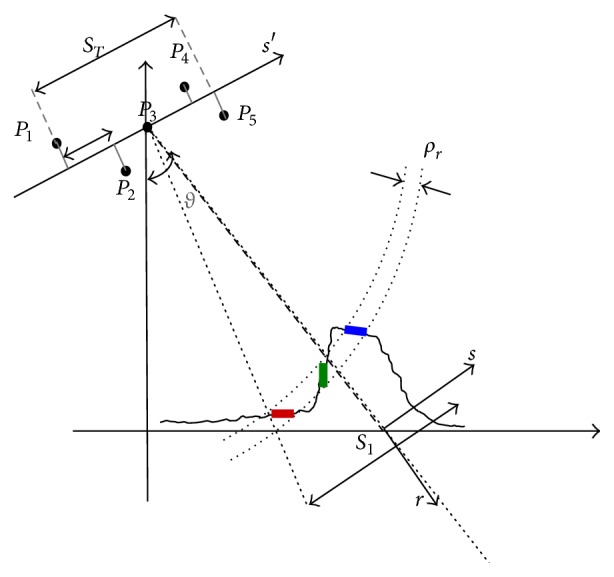
Multipass SAR geometry in the range-elevation plane (case *M* = 5).

SAR tomography allows the reconstruction of the reflectivity profile of the observed scene along the coordinates of range *r*, azimuth *x*, and elevation *s*. In order to estimate the 3D reflectivity function *γ*(*r*, *x*, *s*), a stack of *M* range-azimuth focused images of the same scene is collected with slightly different view angles. The estimation of the reflectivity profile along elevation can be performed by fixing the range azimuth pixel and considering only the dependence on the elevation coordinate. Let us denote with *u*(*s*_*k*_′) a fixed range-azimuth pixel of the *k*th image acquired along the orbit with orthogonal baseline *s*_*k*_′ (see Figure 1), after image registration and atmospheric phase errors compensation. The multipass acquisitions in a fixed range-azimuth pixel can be expressed as [4]

(1) $$\begin{array}{c} u=\Phi \gamma +w, \end{array}$$

where **u** = [*u*(*s*_1_′),…,*u*(*s*_*M*_′)]^*T*^ is the *M* × 1 measurement vector, **γ** = [*γ*(*s*_1_),…,*γ*(*s*_*N*_)]^*T*^ is the unknown *N* × 1 ground reflectivity profile sampled in the elevation values *s*_*k*_ = *k*Δ*s*, **w** is the additive noise, and Φ is *M* × *N* matrix related to the acquisition geometry, whose generic element with index *kl* is given by

(2) $$\begin{array}{c} {\left\{\Phi \right\}}_{kl}=e^{j\left(4\pi /\lambda R_{0}\right)s_{k}^{\prime }s_{l}}, \end{array}$$

with *λ* is the operating wavelength and *R*_0_ is the distance between the centre of the scene and a reference antenna position.

The recovery of the vector **γ** from the measurements **u** can be performed by inverting (1). When performing this inversion, the following problems have to be faced [4]: (1) the number of acquisitions *M* is usually lower than the number of reflectivity samples to be estimated *N*, so that the problem is undetermined; (2) the *M* acquisitions are not uniformly spaced, so that spurious side-lobes and outliers can be present in the solution recovered. To regularize this ill-posed problem, truncated singular value decomposition (T-SVD) [1] can be applied. However, it does not provide satisfying reconstructions when *M* is noticeably smaller than *N*. An alternative way for regularizing the problem consists in exploiting the assumption that only few scatterers at different elevations can lay in the same range azimuth resolution cell (see Figure 1), so that **γ** is a *K*-sparse vector, with *K* being small, and CS theory can be applied. It states that, under certain conditions, it is possible to recover the *K* largest elements of **γ** from a set of *M* = *O*(*K*log⁡(*N*/*K*)) measurements **u**, by solving an *ℓ*_1_-norm minimization problem and achieving superresolution [4].

A reduction of the number of measurements required for achieving a reliable solution can be obtained when a full reconstruction of the signal **γ** is not required. In SAR tomographic applications, this happens when we are interested only in the localization of multiple coherent scatterers and not in their intensity. In this case, the problem amounts in estimating only the position of the *K* nonzero elements in the sparse unknown vector **γ** (of size *N*) from a noisy measurement vector **u** (of size *M*, with *M* < *N* and *M* > *K*) and is commonly referred as support detection. Once the support set is known, system (1) is simplified to an overdetermined system, which can be solved by conventional approaches.

## 3. Support Detection from Compressive Measurements

In this section, we want to analyze some methods for estimating the position of multiple scatterers, by means of support detection techniques.

The detection of the support of a sparse signal can be addressed using different methods.

### 3.1. Maximum Likelihood Detection

Since **γ** in (1) is an unknown deterministic vector, the probability of error in detecting the support is minimized by maximum likelihood (ML) detection. In the Gaussian noise assumption, the ML detector finds the *K*-dimensional subspace spanned by columns of Φ containing the maximum energy of **u** [13]. When the minimum signal to noise ratio (SNR) of the nonzero components tends to infinity, the number of measurements required for obtaining a negligible probability of error in the support detection scales to *M* = *O*(*K*), which is the minimum number of measurements in the noise free case. This method has the best compressive performance but has the drawback to be computationally heavy when *K* increases.

### 3.2. LASSO Detection

A practical method to detect the position of the nonzero elements of the unknown sparse signal is LASSO [14], also called basis pursuit denoising [15]. The LASSO estimate of **γ** is obtained by solving the convex optimization:

(3) $$\begin{array}{c} \hat{\gamma }=\underset{\gamma }{\arg\;\min}\left({\left\|u-\Phi \gamma \right\|}_{2}^{2}+\mu {\left\|\gamma \right\|}_{1}\right), \end{array}$$

where *μ* > 0 is an algorithm parameter that encourages sparsity in the solution. The position of the negligible elements of the signal reconstruction can be used for the support estimation. However, the problem of identifying a thresholding criterion is to be faced. A major problem with thresholding LASSO is that their performances “saturate” with high SNR. That is, even as the SNR scales to infinity, the minimum number of measurements does not scale as *M* = *O*(*K*).

In [16], necessary and sufficient conditions for asymptotic reliable detection with LASSO, for *M*, *N*, and *K* tending to infinity and SNR growing unboundedly, are given. Specifically, the scaling *M* > 2*K* · log⁡(*N* − *K*) + *K* + 1 is both necessary and sufficient for asymptotic reliable detection.

In contrast, optimal ML detection techniques can achieve scaling *M* = *O*(*K*), when the SNR is sufficiently high [16].

### 3.3. OMP and SequOMP Approaches

Another common approach to support detection is the orthogonal matching pursuit (OMP) algorithm [17]. It was analyzed in [18] in a setting with no noise and generalized to the case with noise in [19]. The result is very similar to condition found for LASSO: if *M*, *N*, *K*, and SNR of the weakest scatterer tend to infinity, a sufficient condition for asymptotic reliable detection is *M* > 2*K* · log⁡(*N* − *K*).

Then, also in this case, even as SNR scales to infinity, the minimum number of measurements does not scale as *M* = *O*(*K*).

The results summarized above suggest a performance gap between ML detection and algorithms like LASSO and OMP, especially when SNR is high. In particular, as SNR increases, the performance of these methods saturates at scaling in the number of measurements that can be significantly higher than that for ML.

A more practical method is a simplified version of OMP, called sequential OMP (SequOMP) [9], which under favorable conditions exhibits a performance which does not saturate at high SNR.

SequOMP is a one-pass version of the OMP algorithm, since it is identical to the standard OMP algorithm of [17] except that SequOMP passes through the data only once, in a fixed order, and is computationally simpler than standard OMP. SequOMP generally has worse performance than standard OMP, but it is much simpler. Moreover, in [9] it is shown that this simple algorithm, when used in conjunction with known conditional ranks, can achieve a fundamentally better scaling at high SNRs than LASSO and OMP. In particular, it has been shown that when the power orders of the nonzero elements are known and the signal-to-noise ratio (SNR) is high, the SequOMP algorithm exhibits a scaling in the minimum number of measurements that is within a constant factor of the more sophisticated LASSO and OMP algorithms [9]. Moreover, when the power profile can be optimized, SequOMP can achieve measurement scaling that is within a constant factor of ML detection. This scaling is better than the best known sufficient conditions for LASSO and OMP [9].

When the knowledge of conditional rank of signal components is not available, SequOMP has a performance worse than OMP and LASSO but exhibits a noticeably lower complexity.

## 4. GRLT Support Detection from Compressive Measurements

The methods presented in the previous section are not Constant False Alarm Rate detection approaches. In this section, we present a CFAR approach, using a sequential GLRT for support detection, based on ML estimation. This approach is compared with the SequOMP, adapted in such a way to have a Constant False Alarm Rate.

The detection problem amounts to distinguish *K*_max_ following statistical hypotheses *H*_0_, *H*_1_,…, *H*_*K*_max__, defined as follows:

(4) $$\begin{array}{c} H_{0}\text{: }u=w\;\text{absence of scatterers},\\ H_{k}\text{: }u=\Phi \gamma +w\;\text{presence of}\;\;k\text{ scatterers}, \end{array}$$

where **γ** is a *k*-sparse vector with *k* ≤ *K*_max_, with *K*_max_ the maximum order of sparsity, that is supposed to be a priori known.

The noise vector **w** can be assumed as circularly symmetric complex (or proper complex) Gaussian vector, with uncorrelated samples and mutually uncorrelated real and imaginary parts, with zero-mean and same variance *σ*_*W*_^2^/2.

When the scatterers are absent (hypothesis *H*_0_), **u** is a circularly symmetric Gaussian random vector with zero-mean and covariance matrix **C** = *σ*_*W*_^2^ **I**, with **I** being the identity matrix, while when the scatterers are present, **u** is a circularly symmetric Gaussian random vector with the same covariance matrix and mean being equal to Φ**γ**.

Probability of detection *P*_*D*_ and false alarm *P*_FA_ can be defined as follows:

(5) $$\begin{array}{c} P_{D}=\frac{1}{K_{\max}}\overset{K_{\max}}{\underset{k=1}{\sum }}P\left(H_{k}\;|\;H_{k}\right),\\ P_{\text{FA}}=1-P\left(H_{0}\;|\;H_{0}\right). \end{array}$$

In 3D SAR tomography, the number of scatterers *K*, the vector **γ** and its support *Ω*_*K*_ (positions of the samples different from zero), and the noise variance *σ*_*W*_^2^ are usually unknown. We only suppose that, on the basis of geometrical considerations, *K*_max⁡_ can be assumed known. Then a suboptimal GLRT test can be applied [20].

Since the hypotheses *H*_0_, *H*_1_,…, *H*_*K*_max__ have a hierarchical structure, the multiple hypothesis testing can be performed by means of a sequence of *K*_max_ steps, each one applying a binary hypothesis test between the hypotheses *H*_*k*−1_ and *H*_≥*k*_, which means deciding between *k* − 1 scatterers or at least *k* scatterers. The statistical test applied at the *k*th step of the sequence is obtained by generalizing the two steps test proposed in [10]:

(6) $$\begin{array}{c} \Lambda_{k}\left(u\right)=\frac{\min_{\Omega_{k-1}}\left[u^{H}\Pi_{\Omega_{k-1}}^{⊥}u\right]}{\min_{\Omega_{K_{\max}}}\left[u^{H}\Pi_{\Omega_{K_{\max}}}^{⊥}u\right]}\overset{H_{k-1}}{\underset{H_{\geq k}}{≶}}\beta_{k} \end{array}$$

with

(7) $$\begin{array}{c} \Pi_{\Omega_{k}}^{⊥}=I-\Phi_{\Omega_{k}}{\left(\Phi_{\Omega_{k}}^{H}\Phi_{\Omega_{k}}\right)}^{-1}\Phi_{\Omega_{k}}^{H}, \end{array}$$

where Φ_*Ω*_*k*__ = [**ϕ**_*i*_1__,…, **ϕ**_*i*_*k*__] is the matrix of size *M* × *k* obtained by extracting *k* columns **ϕ**_*i*_, with *i* = 1,…, *k*, of Φ, with *k* = 1,…, *K*_max_. *Ω*_*k*_ is the set of all possible support of cardinality going from *k* to *K*_max_, with *Ω*_0_ the empty set and Φ_*Ω*_0__ = 0, and Π_*Ω*_*k*__^⊥^ is the projector onto the orthogonal complement to the subspace spanned by Φ_*Ω*_*k*__.

Note that the sequential test described by (6) with *k* = 0,…, *K*_max_ − 1 detects the scatterers with different intensities in an order going from strongest to weakest, thanks to the minimization operation at the numerator of (6). Then, the first scatterer to be detected (if there are more than one) at the step *k* = 1 is the one that is responding with the highest intensity.

The thresholds *β*_*k*_ can be derived with a CFAR approach, and the corresponding receiver operating characteristic (ROC) curve can be numerically evaluated by means of Monte Carlo simulation, fixing the value of *P*_FA_ for the first step, and of the probability of false detection *P*_FD_*k*__for the other steps, where

(8) $$\begin{array}{c} P_{{\text{FD}}_{k}}=\overset{K_{\max}}{\underset{l=k}{\sum }}P\left(H_{l}\;|\;H_{k-1}\right). \end{array}$$

## 5. Numerical Results

To analyze the compressive capability of the proposed detection scheme, we consider the problem of detecting one or two scatterers (*K*_max⁡_ = 2) lying in the same range azimuth resolution cell, with the same amplitude, using 25 and 13 tomographic data sets simulated using COSMO-SkyMed (CSK) parameters (see Table 1). We compare the detection performance obtained with different SNR in the range 0–10 dB, for *P*_FA_  =  10^−3^ and for a scatterers separation distance *D*_*s*_ = 2*ρ*_*s*_, with *ρ*_*s*_ the nominal elevation resolution related to the maximum orthogonal baseline extent *S*_*T*_ [1]:

(9) $$\begin{array}{c} \rho_{s}=\frac{\lambda R_{0}}{2S_{T}}. \end{array}$$

In this case, the proposed GRLT (6) is applied in two steps.

We compare the proposed method with the SequOMP approach. The SequOMP algorithm has been implemented using a threshold providing the desired *P*_FA_ [14].

**Figure 2:**
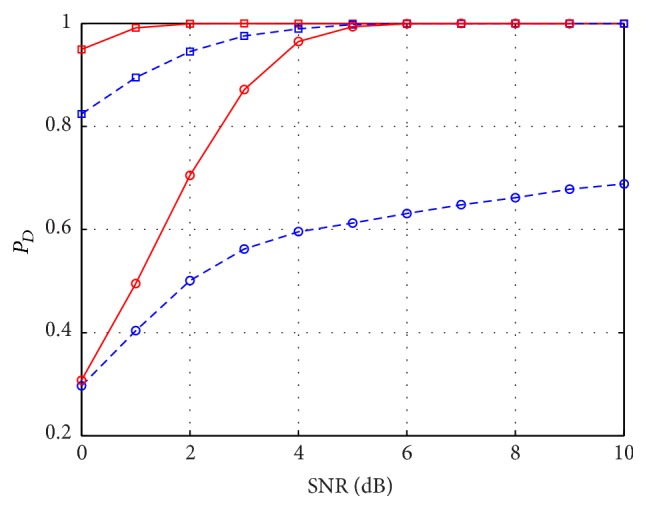
*P*_*D*_ versus SNR for SequOMP detector (blue dashed line) and Sup-GLRT detector (red solid line), in the case of two scatterers of same amplitude and at a distance *D*_*s*_ = 2*ρ*_*s*_, with *P*_FA_ = 10^−3^, *M* = 13 (circle markers), and *M* = 25 (square markers).

In order to compare Sup-GLRT with SequOMP, the probability of false alarm in both approaches is defined according to (5), that is, the probability of the event that the algorithm falsely detects a support different from the empty set in the hypothesis *H*_0_. We evaluated the detection performance according to definition (4) of probability of detection, *P*_*D*_ = 0.5[*P*(*H*_1_/*H*_1_) + *P*(*H*_2_/*H*_2_)], to fairly compare the two approaches. We report *P*_*D*_ obtained using the two approaches with respect to SNR and for *M* = 25 and *M* = 13, to see the effect of decreasing the number of measurements. In particular, *P*_*D*_ relative to the SequOMP is reported in Figure 2 with blue dashed line and *P*_*D*_ relative to Sup-GLRT in red solid line, using square marker for *M* = 25 and the circle markers for *M* = 13. It can be seen that Sup-GLRT outperforms SequOMP in both cases, showing its robustness with respect to the number of measurements.

**Figure 3:**
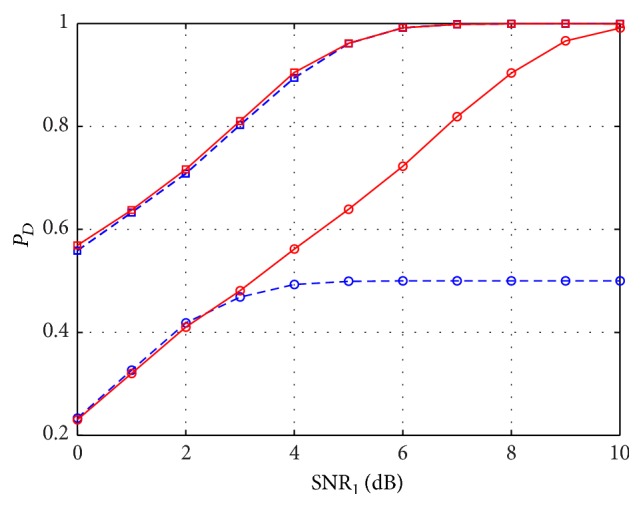
*P*_*D*_ versus SNR for SequOMP detector (blue dashed line) and Sup-GLRT detector (red solid line), in the case of two scatterers of different amplitude with 3 dB SNR difference (SNR_1_ = SNR_2_ + 3 dB) and at a separation distance *D*_*s*_ = 2*ρ*_*s*_, with *P*_FA_ = 10^−3^, *M* = 13 (circle markers), and *M* = 25 (square markers).

In Figure 3, we report the results of the same experiment considering again two scatterers at a distance *D*_*s*_ = 2*ρ*_*s*_, but with different SNRs. the strongest one has SNR_1_ = SNR_2_ + 3 dB, where SNR_2_ corresponds to the weaker scatterer. In this case, it can be noted that the performance is equivalent for *M* = 25, while if the number of measurements is reduced, Sup-GLRT outperforms SequOMP. In SequOMP implementation, a blind detection order has been followed, since it is not possible to assume any a priori information on the scatterers power order.

**Figure 4:**
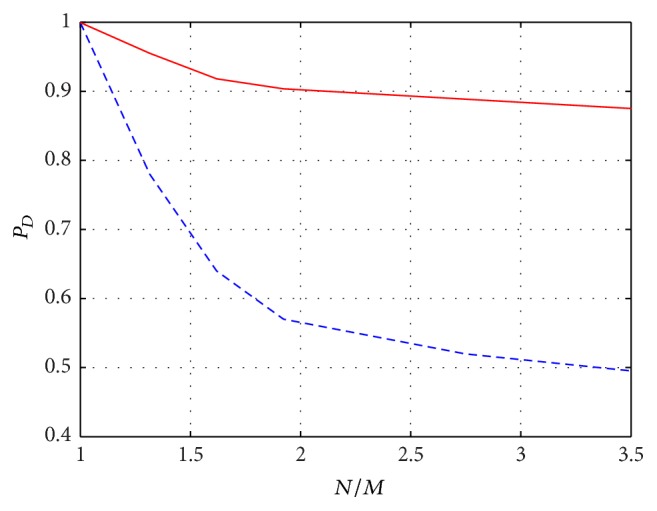
*P*_*D*_ versus *N*/*M* for SequOMP detector (blue dashed line) and Sup-GLRT detector (red solid line), in the case of two scatterers of different amplitude with 3 dB SNR difference (SNR_1_ = 8 dB and SNR_2_ = 5 dB), at a separation distance *D*_*s*_ = 2*ρ*_*s*_ and for *P*_FA_ = 10^−3^.

Eventually, we report in Figure 4 *P*_*D*_ relative to SequOMP and Sup-GLRT, considering a strongest scatterer with SNR_1_ = 8 dB and a weaker scatterer with SNR_2_ = 5 dB, and for a varying ratio *N*/*M*, in order to emphasize the impact of compressing the measurements. It can be noted that when *N*/*M* increases (i.e., *M* decreases), *P*_*D*_ obtained with Sup-GLRT is higher than the one obtained with SequOMP, confirming the higher robustness of the method proposed when dealing with compressed measurements.

## 6. Conclusions

In this paper we analyze the problem of identifying multiple scatterers lying in the same range azimuth resolution cell from a compressive number of multibaseline SAR images. We followed a support detection approach, which adapts well to the sparse unknown signal. Different support detection methods have been considered and the performance of two different schemes has been investigated: a GLRT based support detection and the SequOMP techniques. Preliminary results on simulated data show that the first one is more robust with respect to the reduction of the number of measurements, since it allows a higher probability of detection for a given probability of false alarm and a given number of measurements. It has been considered the detection and localization of two scatterers responding with the same intensities and of two scatterers responding with different intensities. Moreover, different ratios *N*/*M* have been considered, in order to show the measurement scaling behavior of the two detectors. The results of numerical simulations show that Sup-GLRT outperforms SequOMP as far as compressive measurement capability is concerned.

## Figures and Tables

**Table 1:** 

COSMO-SkyMed parameters	
Wavelength	0.031 m
View angle	35°
Range distance	620 Km
